# DEEPSEN: a convolutional neural network based method for super-enhancer prediction

**DOI:** 10.1186/s12859-019-3180-z

**Published:** 2019-12-24

**Authors:** Hongda Bu, Jiaqi Hao, Yanglan Gan, Shuigeng Zhou, Jihong Guan

**Affiliations:** 1 Department of Computer Science and Technology, Tongji University, 4800 Cao’an Road, Shanghai, 201804 China; 2 Shanghai Key Lab of Intelligent Information Processing, and School of Computer Science, Fudan University, 220 Handan Road, Shanghai, 200433 China; 3 School of Computer Science and Technology, Donghua University, 2999 North Renmin Road, Shanghai, 201620 China

**Keywords:** Super-enhancer prediction, Deep learning, Convolutional neural network

## Abstract

**Background:**

Super-enhancers (SEs) are clusters of transcriptional active enhancers, which dictate the expression of genes defining cell identity and play an important role in the development and progression of tumors and other diseases. Many key cancer oncogenes are driven by super-enhancers, and the mutations associated with common diseases such as Alzheimer’s disease are significantly enriched with super-enhancers. Super-enhancers have shown great potential for the identification of key oncogenes and the discovery of disease-associated mutational sites.

**Results:**

In this paper, we propose a new computational method called DEEPSEN for predicting super-enhancers based on convolutional neural network. The proposed method integrates 36 kinds of features. Compared with existing approaches, our method performs better and can be used for genome-wide prediction of super-enhancers. Besides, we screen important features for predicting super-enhancers.

**Conclusion:**

Convolutional neural network is effective in boosting the performance of super-enhancer prediction.

## Background

Numerous transcriptional factors combine with enhancers to regulate gene expression through recruiting transcriptional coactivator and RNA polymerase to target gene [1]. The term ‘enhancer’ was first introduced to describe the effects of SV40 DNA on the ectopic expression of a cloned rabbit *β* globin gene. The SV40 DNA elements activated transcription at a distance and independently of their orientation concerning the target gene [2]. Enhancer activation often coincides with DNase I hypersensitivity of these regions and with specific post-translational modifications of adjacent nucleosomes [3]. Direct interaction or looping between enhancers and the promoters of target genes has been observed and might be critical to enhancer function [4, 5]. Recently, advances in DNA sequencing technology, such as Chromatin Immunoprecipitation sequencing(ChIP-seq) and DNase I hypersensitivity sites sequencing(DNase-seq) have enabled the discovery of putative mammalian enhancers on a genome-wide scale [6–10].

The concept of super-enhancers was proposed by Richard A.Young based on the research on enhancers, which is described as a class of regulatory regions with unusually strong enrichment for the binding of transcriptional coactivators, specifically Mediator (Med1) [11, 12]. In mouse embryonic stem cells (mESCs), super-enhancers were defined in the following way [12]: 1) Sites bound by all three master regulators, Oct4, Sox2 and Nanog, according to ChIP-seq, were considered enhancers; 2) Enhancers within 12.5 kb of each other were stitched to define a single entity spanning a genomic region; 3) The stitched enhancer entities and the remaining individual enhancers (those without a neighboring enhancer within 12.5 kb) were then ranked by the total background-normalized level of the Med1 signal within the genomic region. A small proportion (less than 3%) of these enhancer regions contained Med1 levels above a cutoff was designated as super-enhancers. The remaining enhancer regions were considered ‘normal’ enhancers. Super-enhancers tend to span large genomic regions, whose median size generally an order of magnitude larger than that of normal enhancers (in mESCs, 8667 bp versus 703 bp) [11–13]. Relative to Med1, a number of factors generally associated with enhancer activity show enrichment at super-enhancers relative to normal enhancers. These factors include RNA polymerase II (Pol II), RNA from transcribed enhancer loci (eRNA), the histone acetyltransferases p300 and CBP, chromatin factors such as cohesin, the histone modifications H3K27ac, H3K4me2 and H3K4me1, and increased chromatin accessibility as measured by DNase-seq. Because of these cross-correlations, super-enhancers might be identified by many of these features [11].

Since super-enhancers influence various biological processes, the identification of super-enhancers becomes an urgent research issue. BRD4, a member of the BET protein family, was used to distinguish super-enhancers from typical enhancers as it is highly correlated with MED1 [13]. H3K27ac was extensively used to create a catalog of super-enhancers across 86 different human cell-types and tissues due to its availability [11]. Other studies used the coactivator protein P300 to define super-enhancers [14, 15] However, the knowledge about these factors’ ability to define a set of super-enhancers in a particular cell-type and their relative and combinatorial importance remains limited. Master transcriptional factors that might form super-enhancers domains are largely unknown for most cell-types, while performing ChIP-seq for the Mediator complex is difficult and costly. However, there are no predictive models that integrate various types of data to predict super-enhancers and their constituents (enhancers within super-enhancer). Besides, to what degree these features influence on super-enhancers remains unknown.

Predicting super-enhancers based on machine learning remains nearly blank in the literature. The only work was done by Khan and Zhang [16]. They used six different machine learning models, including Random Forest, linear SVM, KNN, AdaBoost, Naive Bayes and Decision Tree. Chromatin, transcription factors and sequence-specific features were used to train these models individually, which were evaluated by 10-fold cross-validation. With the rise of deep learning (DL) techniques, many researchers applied state-of-art DL methods to bioinformatics problems. In DEEPBIND [17], Alipanahi et al. described the use of a deep learning strategy to calculate protein-nucleic acid interactions from diverse experimental data sets. Their results showed DL’s applicability in bioinformatics and improved prediction power over traditional methods. Besides, Zhou et al. developed a deep-learning based algorithmic framework, named DeepSEA, which learns a regulatory sequence code from large-scale chromatin-profiling data in order to predict the noncoding variants effects [18].

In this work, we proposed a novel approach to solving the problem of super-enhancer prediction based on convolutional neural networks (CNNs). This method is called DEEPSEN. We constructed different structures of CNN to discover which kind of structure is more appropriate for the problem. For each network structure, we did fine-tuning to find out the best parameter set and to avoid overfitting. Furthermore, we did feature ranking and found out the significance of features for super-enhancers prediction. Our experimental results demonstrate that DEEPSEN outperforms the existing super-enhancer prediction model.

## Methods

### Datasets

Similar to Aziz Khan [16], we obtained 32 publicly available ChIP-seq and DNase-seq datasets of mouse embryonic stem cells (mESC) from Gene Expression Ominibus (GEO). These data cover four histone modifications (H3K27ac, H3K4me1, H3K4me3 and H3K9me3), DNA hypersensitive site (DNaseI), RNA polymeraseII (Pol II), transcriptional co-activating proteins (p300 and CBP), P-TFEb subunit (Cdk9), sub-units of Mediator complex (Med1, Med12 and Cdk8), chromatin regulators (Brg1, Brd4 and Chd7), Cohesin (Smc1 and Nipbl), subunits of Lsd1-NuRD complex (Lsd1 and Mi2b) and 11 transcription factors (Oct4, Sox2, Nanog, Esrrb, Klf4, Tcfcp2l1, Prdm14, Nr5a2, Smad3, Stat3 and Tcf3). Table 1 shows the datasets used in this paper.

**Table 1:** Datasets used in this paper

Data Type	Data Name	GEO ID
Transcription factors	Oct4, Sox2, Nanog, Esrrb, Klf4, Smad3, Tcfcp2l1, Prdm14, Stat3, Tcf3, Nr5a2	GSE44286, GSM288355, GSM288354, GSM623989, GSM53954
Mediator complex	MED1	GSM560348,GSM560345
Histone modifications	H3K27ac, H3K4me1, H3K4me3, H3K9me3	GSM594579, GSM281695, GSM307149, GSM18371
RNA polymerase	RNA Pol	GSM318444
Hypersensitive site	DNaseI	GSM1014154
Co-activators	p300, CBP	GSM918750,GSM1246866
Chromatin regulators	Brg1, Brd4, Chd7	GSM896923, GSM937540, GSM558674
Cohesion	Nipbl, Smc1	GSM560350,GSM560342
Mediator complex	MED12	GSM560348,GSM560345
Lsd1-NuRD complex	Lsd1, Mi2b	GSM687282,GSM687284

We used MED1 signal to define super-enhancers as described in ROSE [12]. We selected transcriptional enriched regions as the training samples. Thus, we obtained 11100 samples with 36 kinds of features. Among them, 1119 are positive samples and 9981 are negative ones.

### Pipeline of the dEEPSEN method

**Fig. 1:**
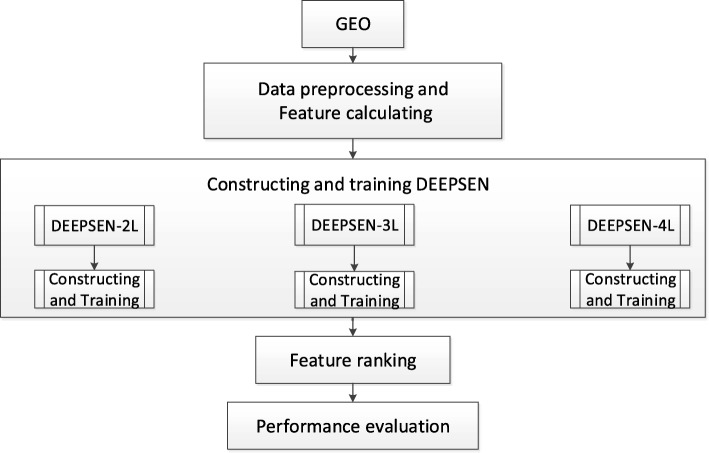
The pipeline of DEEPSEN. The data we used were from GEO. Firstly, we do data preprocessing and feature calculation. Secondly, we construct three models with different numbers of convolutional layers and train them. Thirdly, we evaluate Pearson correlation coefficient to rank the features for predicting super-enhancers. Finally, we do performance evaluation and analysis

Based on convolutional neural network (CNN), we proposed a novel approach named DEEPSEN to predict super enhancers on genome scale. Fig. 1 illustrates the pipeline of the DEEPSEN method. It consists of three major steps:

1. Data preprocessing and feature calculation. 36 kinds of features were used to represent super-enhancers, including DNA sequence compositional features, histone modifications, transcriptional factors, RNA polymeraseII, hypersensitive site, co-activators, chromatin regulators, cohesion, mediator complex, mediator complex, and Lsd1-NuRD complex.
2. Constructing and training DEEPSEN. First, we built three models with different numbers of convolutional layers. Then, we trained each model using the back propagation (BP) algorithm [19] and stochastic gradient descent optimization algorithm. Furthermore, we did parameter tuning and validated each model using 5-fold cross-validation.
3. Feature ranking. We evaluated each feature’s contribution to the identification of super-enhancers.

In what follows, we elaborate the process of super-enhancer prediction step by step.

### Data preprocessing and feature selection

Firstly, we aligned the original ChIP-seq reads to mouse genome-build mm9 with bowtie 0.12.9 [20]. As a result, we got the start and end positions of each read. Secondly, with these positions and the help of bamtoGFF, we calculated the read densities of samples, including super-enhancers and normal enhancers, and normalized these densities. Thirdly, we evaluated the binding affinity scores of all the samples with DNA binding motif information. Finally, we combined the calculated read densities and the binding affinity scores to get the final training data.

### Constructing and training dEEPSEN

#### The structure of dEEPSEN

**Fig. 2:**
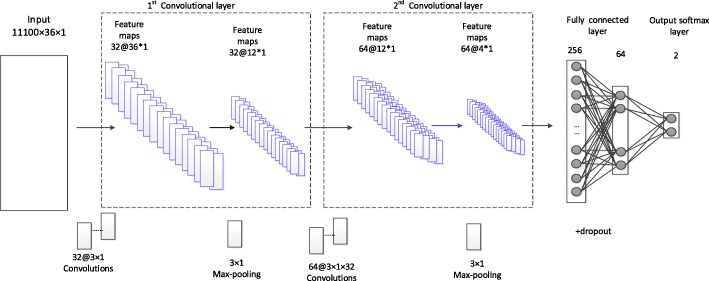
The architecture of DEEPSEN-2L. DEEPSEN-2L consists of the input layer, the 1st convolutional layer (including the 1st max-Pooling), the 2nd convolutional layer (including the 2nd max-Pooling), fully connected layer (including dropout), and output softmax layer

Figure 2 shows the architecture of a DEEPSEN classifier, which consists of the *input layer* (the 1st convolutional layer, including max-pooling), the *2nd convolutional layer* (including max-pooling),..., the *fully connected layers*, and the *output layer*.

The convolutional layer contains two steps: convolution step and pooling step. The convolution step uses multiple convolutional kernels to do convolution operation on the input data. A max-pooling operation often follows a convolution step to output a local maximal value of the respective convolutional outputs. The convolution operation learns to recognize relevant patterns of the input. The function of max-pooling is to reduce parameters to abstract the features learned in the proceeding layers. An activation function is usually used after each layer, which is nonlinear to guarantee the nonlinearity of the whole model. Here, we used the rectified linear unit(ReLU) function:

1 $$ ReLU(x) = max(0,x)  $$

The subsequent convolutional layers capture the relationships of the features extracted from the proceeding layers to obtain high-level features. Finally, the fully connected layer with dropout transforms the input into probability distribution through the softmax function:

2 $$ f_{i}(z) = \frac{\mathrm{e}^{z_{i}}}{\sum_{j}\mathrm{e}^{z_{j}}}  $$

The parameter details of the architecture are described in Table 1. We take the model consisting of 2 convolutional layers as the example. The input layer is a N ×36×1 matrix, where *N* is the number of samples that is set to 11100 in our experiments. The first convolutional layer contains 32 kernels of shape 3 ×1 with the stride of 1 using the same padding so that the size does not change during convolution operation with. The output of the first layer includes 32 feature maps of size 36 ×1. Next is the first pooling layer of size 3 ×1, which means that we remain only the maximum value among every three values to reduce the dimensions and make the model robust. The second convolutional layer has 64 kernels, each of which is 3 ×1×32, and its output includes 64 feature maps of size 12 ×1. The 2nd pooling layer uses 3 ×1 max-pooling, and its output contains 64 feature maps of size 4 ×1, that is, 64*4=256 nodes. Following is the fully connected layer with 256 input nodes and 64 output nodes. We used dropout method [21] in the fully connected layer to delete some nodes randomly for controlling over-fitting. The detailed structure of DEEPSEN that contains two convolutional layers is presented in Table 2. Besides the DEEPSEN with two convolutional layers, we also constructed DEEPSEN predictors with three convolutional layers and four convolutional layers. The details are presented in Tables 3 and 4, respectively.

**Table 2:** The structure of DEEPSEN-2L

Layer	Size	Output Shape
Input		36 ×1
Convo1	32 ×3×1	32 ×36×1
Pool1	1 ×3	32 ×12×1
Convo2	64 ×3×1×32	64 ×12×1
Pool2	1 ×3	64 ×4×1
Full-connected	256	64
softmax	64	2

**Table 3:** The structure of DEEPSEN-3L

Layer	Size	Output Shape
Input		36 ×1
Convo1	32 ×3×1	32 ×36×1
Pool1	1 ×3	32 ×12×1
Convo2	64 ×3×1×32	64 ×12×1
Pool2	1 ×3	64 ×4×1
Convo3	128 ×3×1×64	128 ×4×1
Pool3	1 ×2	128 ×2×1
Full-connected	256	64
softmax	64	2

**Table 4:** The structure of DEEPSEN-4L

Layer	Size	Output Shape
Input		36 ×1
Convo1	32 ×3×1	32 ×36×1
Pool1	1 ×3	32 ×12×1
Convo2	64 ×3×1×32	64 ×12×1
Pool2	1 ×3	64 ×4×1
Convo3	128 ×3×1×64	128 ×4×1
Pool3	1 ×2	128 ×2×1
Convo4	256 ×3×1×128	256 ×2×1
Pool4	1 ×2	256 ×1×1
Full-connected	256	64
softmax	64	2

The major difference between the CNN based models and previous models lies in that CNN can learn to recognize relevant patterns of input by updating the network during training. Therefore, the advantage of CNN based models is the ability to learn complicated features from large-scale datasets in an adaptive manner.

#### The training of dEEPSEN

We used the cross entropy loss function, which is as follows:

3 $$ {J(\theta)=-\frac{1}{m}\sum_{i=1}^{m}y^{i}\log(h_{\theta}(x^{i}))+(1-y^{i})\log(1-h_{\theta}(x^{i}))}  $$

where *θ* is the parameter set, m is the amount of samples, y ^*i*^ is the label of x ^*i*^, h _*θ*_(x ^*i*^) is the predicted label of x ^*i*^. Parameters were randomly initialized. The data was processed from the input layer to the output layer, and back propagation [19] and stochastic gradient descent algorithms were used to update the network parameters to minimize the cost function. Each epoch contains forward propagation, loss calculation, back propagation and parameter refreshing. The detailed training steps are as follows:

1. Initializing the parameters randomly.
2. Feeding the training data to the input layer.
3. Doing convolution operation and max-pooling operation in each conventional layer
4. Using the output of the last convolutional layer as the input of fully connected layer to obtain the result of the output layer
5. Evaluating the cost function and doing Adam optimization [22] using the BP algorithm [19] to refresh the parameters
6. Repeating step 2 to step 5 (one epoch) to recalculate the cost function until the desirable number of iterations is reached.

### Feature ranking

In our models, we integrated 36 different features to predict super enhancers, including H3K27ac, H3K4me1, H3K4me3, H3K9me3, Brd4, Cdk8, Cdk9, Med12, p300, CBP, Pol2, Lsd1, Brg1, Smc1, Nipbl, Mi2b, CHD7, H- DAC2, HDAC, DNaseI, 4-Oct, Sox2, Nanog, Smad3, Stat3, Tcf3, Esrrb, Klf4, Prdm14, Tcfcp2I1, Nr5a2, AT content, GC content, phastCons, phastConsP, re- peat fraction. To measure the predictive power of each feature, we computed the Pearson correlation coefficient between each feature vector and the output label vector of all test samples. Then, we ranked these features based on the calculated Pearson correlation coefficient.

## Results and discussion

### Parameter tuning

DEEPSEN was implemented on tensorflow [23] with python. To investigate the impact of the number of convolutional layers on prediction performance, we constructed three models with different layers of convolutional neural networks, concretely, two, three and four convolutional layers. For simplification, these models are denoted as DEEPSEN-2L, DEEPSEN-3L and DEEPSEN-4L, respectively.

For each model, although most parameters were tuned automatically in the training process of the convolutional neural networks, there are still some hyper-parameters to be determined. Here, the Adam optimization method [22] was applied. We used grid search to tune the hyper-parameters, including learning rate, the number of epoches and the number of layers. Based on a number of preliminary experiments, we limit the parameters in the following ranges: the number of layers *L*: 2-4 (with stride 1); the number of epoches *e*: 50-150 (with stride 10); learning rate *α*: 10^−5^, 5 ×10^−5^, 10^−4^, 5 ×10^−4^, 10^−3^, 5 ×10^−3^, 10^−2^, 5 ×10^−2^.

**Fig. 3:**
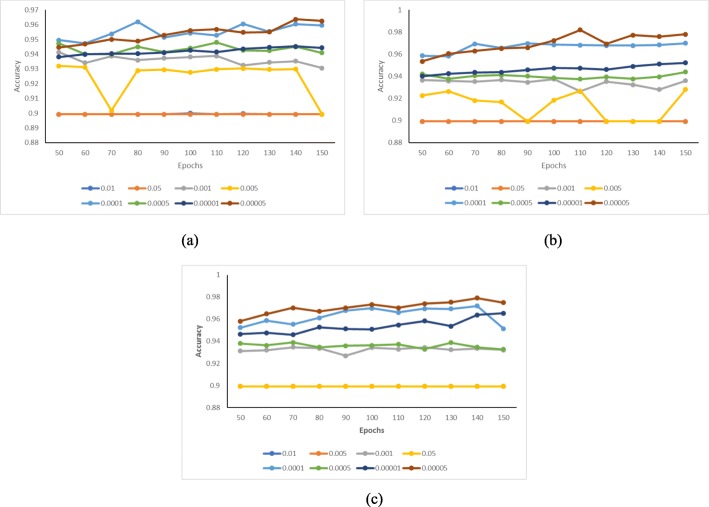
Accuracy results under different parameter sets. **a** Accuracy vs. epochs for different *α* (DEEPSEN-2L); **b** Accuracy vs. epochs for different *α* (DEEPSEN-3L); **c** Accuracy vs. epochs for different ? (DEEPSEN-4L)

We used accuracy as evaluation metric to tune parameters. The results are shown in Fig. 3. For DEEPSEN-2L, when *α* is set between 0.00005 and 0.0001, it achieves better prediction accuracy. Generally, the accuracy increases with the number of epoches (for the number of epoches ≤ 140). We did not choose too large numbers of epoches for the reason of training efficiency. When *α* is set to between 0.01 to 0.05, the accuracy is fixed at 0.9 because *α* is so large that gradient descent algorithm can not perform well, and DEEPSEN-2L predicts all samples as negatives (note that the ratio of negatives over positives is 9). DEEPSEN-3L and DEEPSEN-4L show similar patterns on parameters tuning. Overall, the optimized learning rate is between 5* 10^−4^ and 10^−4^, the optimized number of epoches is between 140-150. With such parameter setting, DEEPSEN-4L achieves a better overall performance. Thus, we chose DEEPSEN-4L as the final model to predict super-enhancers. In what follows, we compare our three models with existing methods in terms of evaluation metrics *precision*, *recall*, *F1* and *AUC*. The definitions of theses evaluation metrics is as follows. In classification task, TP denotes the true positives, FP denotes the false positives, TN denotes the true negatives and FN denotes the false negatives. ROC(Receiver Operating Characteristic) curve describe the relation between FP rate and TP rate, AUC is the area under curve.

4 $$ Precision = \frac{TP}{TP+FP}  $$

5 $$ Recall = \frac{TP}{TP+FN}  $$

6 $$ F1 = \frac{2*Precision*Recall}{Precision+Recall}  $$

### Performance evaluation

**Fig. 4:**
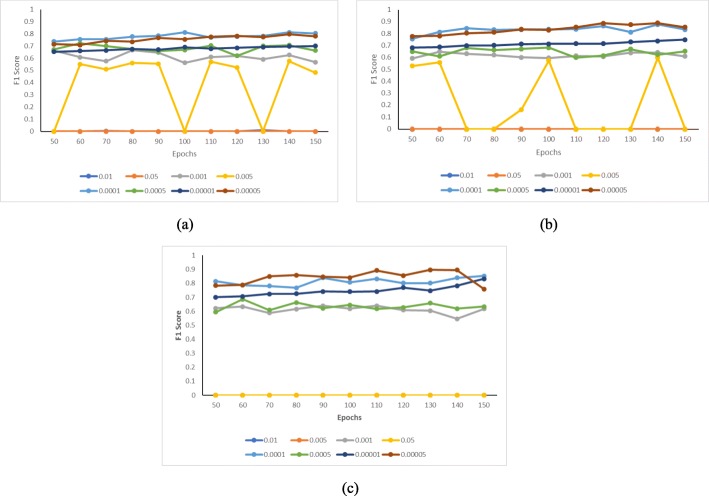
*F1* results under different parameter sets; **a** F1 vs. epochs for different *α* (DEEPSEN-2L). **b** F1 vs. epochs for different *α* (DEEPSEN-3L). **c** F1 vs. epochs for different *α* (DEEPSEN-4L)

The *F*1 values of our three models under different hyper-parameter settings are shown in Fig. 4. For DEEPSEN-2L, the best performance is achieved with *α*=0.0001 and the number of epoches being 140. For DEEPSEN-3L, the best performance is obtained when *α*=0.00005 and the number of epoches is 140. As for DEEPSEN-4L, the best performance comes from *α*=0.00005 and the number of epoches being 130. So we can see that all the three models of DEEPSEN achieve the best *F*1 when *α* is between 0.00005 and 0.0001, and the number of epoches is between 130 and 140. This observation is also noticed on accuracy.

**Fig. 5:**
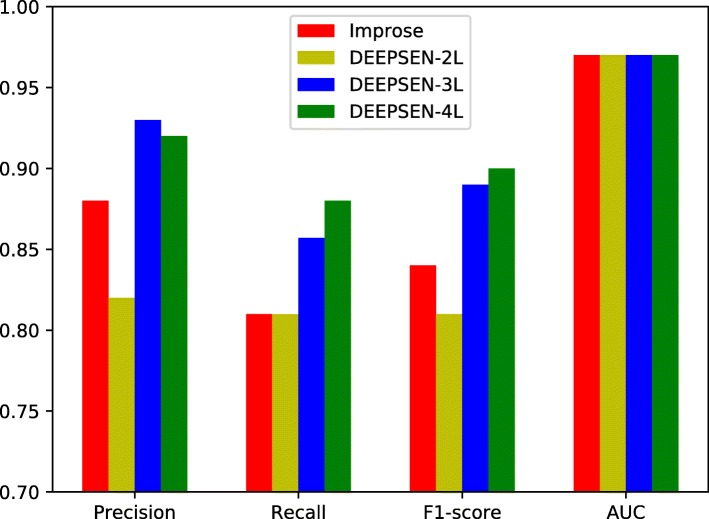
Performance comparison with improse

**Fig. 6:**
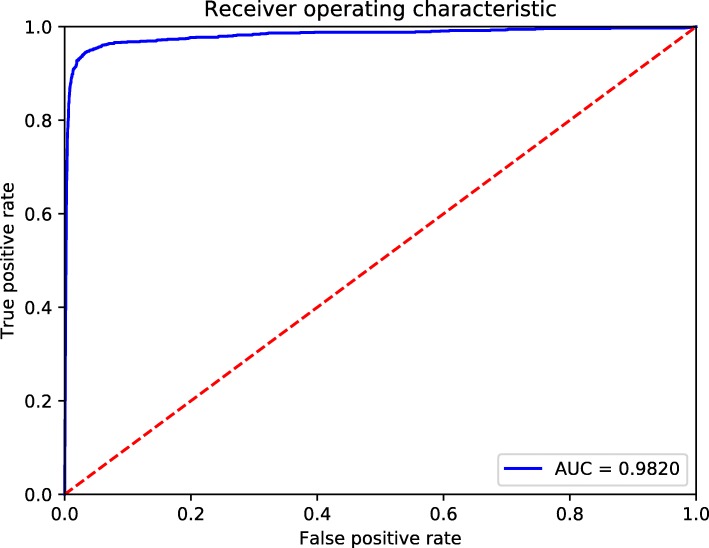
The best ROC curve of DEEPSEN

The performance results of DEEPSEN with different structures are given in Table 5, where the performance results of improse [16] are listed for comparison. We can see that DEEPSEN-3L and DEEPSEN-4L perform better than improse in terms of precision, recall and F1. It demonstrates that the proposed DEEPSEN method outperforms the stat-of-the-art method improse. Figure 5 shows the performance comparison between our models and improse, and Fig. 6 shows the best AUC of DEEPSEN-4L when *α*=0.00005 and the number of epoches is 110.

**Table 5:** Performance comparison with the state-of-the-art method

Method	Precision	Recall	F1-score	AUC
Improse	0.88	0.81	0.84	0.97
DEEPSEN	0.92	0.88	0.90	0.97

### Performance comparison among different features

The results of the first six correlated features are presented in Table 6. The Pearson correlation coefficient indicates the contribution of each feature to prediction performance. For our method, the feature ranking according to Pearson correlation coefficient is: Med12, cdk8, Brd4, Cdk9, P300, H3K27ac, which is roughly similar to the findings of improse. The ranking given by improse is: Brd4, H3K27ac, Cdk8, Cdk9, Med12 and p300.

**Table 6:** The results of feature ranking

Features	Med12	Cdk8	Brd4	Cdk9	p300	H3K27ac
Correlation	0.746	0.731	0.684	0.643	0.618	0.605

## Conclusion

In this paper, we proposed DEEPSEN, a new super-enhancer prediction method based on convolutional neural networks (CNNs). The data from GEO were used to train and test the proposed method. 36 kinds of features, including DNA sequence, histone modifications and TF bindings were integrated to train three models with 2, 3 and 4 convolutional layers. DEEPSEN uses a three-step scheme to construct and train CNN based classifiers. The first step is data preprocesing and feature calculation. The second step is to construct and train DEEPSEN. The third step is feature ranking. Our experimental results show that DEEPSEN outperforms the existing methods. DEEPSEN can be used with high-throughput experimental techniques to improve the accuracy of super-enhancer prediction.

## Data Availability

The data and materials are available at https://github.com/1991Troy/DEEPSEN

## Abbreviations

BP: Back-propagation
ChIP-seq: Chromatin Immunoprecipitation sequencing
CNNs: Convolutional neural networks
DL: Deep learning
DNase-seq: DNase I hypersensitive sites sequencing
eRNA: Enhancer RNA
GEO: Gene Expression O- minibus
KNN: K-nearest neighbors
mESCs: Mouse embryonic stem cells
SEs: Super-enhancers
SVM: Support vector machine
